# The GENOTEND chip: a new tool to analyse gene expression in muscles of beef cattle for beef quality prediction

**DOI:** 10.1186/1746-6148-8-135

**Published:** 2012-08-15

**Authors:** Jean-Francois Hocquette, Carine Bernard-Capel, Veronique Vidal, Beline Jesson, Hubert Levéziel, Gilles Renand, Isabelle Cassar-Malek

**Affiliations:** 1 INRA, UMR1213, Unité de Recherches sur les Herbivores, Theix, Saint-Genès-Champanelle, F-63122, France; 2 VetAgro Sup, UMR1213, Unité de Recherches sur les Herbivores, Saint Genès Champanelle, F-63122, France; 3 Institut de l’Elevage, Service Aptitudes et Sélection des Races Allaitantes, Aubière, F-63170, France; 4 IMAXIO, Biopôle Clermont-Limagne, Saint-Beauzire, F-63360, France; 5 INRA, UMR1061, Unité de Génétique Moléculaire Animale, Limoges, F-87060, France; 6 Université de Limoges, UMR1061, Unité de Génétique Moléculaire Animale, Limoges, F-87060, France; 7 INRA, UMR1313, Génétique Animale et Biologie Intégrative, Jouy-en-Josas, F-78352, France; 8 AgroParisTech, INRA UMR1313, Génétique Animale et Biologie Intégrative, Jouy-en-Josas, F-78352, France

**Keywords:** Predictors, Beef, Tenderness, Array, Gene expression

## Abstract

**Background:**

Previous research programmes have described muscle biochemical traits and gene expression levels associated with beef tenderness. One of our results concerning the *DNAJA1* gene (an Hsp40) was patented. This study aims to confirm the relationships previously identified between two gene families (heat shock proteins and energy metabolism) and beef quality.

**Results:**

We developed an Agilent chip with specific probes for bovine muscular genes. More than 3000 genes involved in muscle biology or meat quality were selected from genetic, proteomic or transcriptomic studies, or from scientific publications. As far as possible, several probes were used for each gene (e.g. 17 probes for *DNAJA1*). RNA from *Longissimus thoracis* muscle samples was hybridised on the chips. Muscles samples were from four groups of Charolais cattle: two groups of young bulls and two groups of steers slaughtered in two different years. Principal component analysis, simple correlation of gene expression levels with tenderness scores, and then multiple regression analysis provided the means to detect the genes within two families (heat shock proteins and energy metabolism) which were the most associated with beef tenderness. For the 25 Charolais young bulls slaughtered in year 1, expression levels of *DNAJA1* and other genes of the HSP family were related to the initial or overall beef tenderness. Similarly, expression levels of genes involved in fat or energy metabolism were related with the initial or overall beef tenderness but in the year 1 and year 2 groups of young bulls only. Generally, the genes individually correlated with tenderness are not consistent across genders and years indicating the strong influence of rearing conditions on muscle characteristics related to beef quality. However, a group of HSP genes, which explained about 40% of the variability in tenderness in the group of 25 young bulls slaughtered in year 1 (considered as the reference group), was validated in the groups of 30 Charolais young bulls slaughtered in year 2, and in the 21 Charolais steers slaughtered in year 1, but not in the group of 19 steers slaughtered in year 2 which differ from the reference group by two factors (gender and year). When the first three groups of animals were analysed together, this subset of genes explained a 4-fold higher proportion of the variability in tenderness than muscle biochemical traits.

**Conclusion:**

This study underlined the relevance of the GENOTEND chip to identify markers of beef quality, mainly by confirming previous results and by detecting other genes of the heat shock family as potential markers of beef quality. However, it was not always possible to extrapolate the relevance of these markers to all animal groups which differ by several factors (such as gender or environmental conditions of production) from the initial population of reference in which these markers were identified.

## Background

There is a need for new knowledge in order to develop animal farming systems that respond to the new and diversified demands of producers and consumers. For producers, it is a question of increasing the yield of meat production per animal, i.e. getting heavy and lean carcasses at the expense of fat to ensure the highest potential income. Consequently, genetic selection has been directed in favour of muscle development in order to produce lean carcasses in Europe. Recent genomic studies have shown that this type of selection may change some muscle characteristics associated with beef quality [1,2]. Bovine genome sequencing has recently revolutionized the genomics research field in an unprecedented manner [3].

Although meat quality traits are moderately heritable, large selection programmes are very difficult to implement to improve beef quality due to the lack of recorded standardized measures of beef quality [4,5]. However, consumers are looking for bovine meat of high and consistent quality. Like the consumers, producers are interested in producing high-quality meat products at the best price.

Research done so far has highlighted the difficulties for control of beef sensory quality. Firstly, meat quality depends on complex and multifactorial biological mechanisms, and the muscle characteristics studied up to now only explain up to one fourth or one third of the variability in meat quality [6]. The origins of high variability of muscle characteristics lie in the genetic potential of the animals and to a greater extent in gene expression within muscles according to environmental factors. Thus, conditions of production (growth path, management, nutrition, etc) interact with the genetic potential of the animals to determine meat quality traits. Secondly, meat ageing plays a key role in the final tenderness of beef. It is thus of paramount importance to increase our knowledge of all these mechanisms in order to better monitor meat quality components.

Intrinsic quality attributes of beef, and especially tenderness, therefore depend partly on the muscle characteristics of live animals, which in turn depend on gene expression. Expression levels of these genes as well as interaction between them can now be accessed through functional genomics (e.g. DNA chips and proteomic tools). Functional genomics is also expected to be helpful for the beef industry that is looking for biological or molecular indicators that would identify live animals with desirable quality attributes, in order to direct them towards the most appropriate production system(s) (for a review, see [7-9]).

The strategy so far has been to identify genes or proteins that are expressed differentially between animals displaying extreme quality attributes without any prior knowledge of the processes involved. Previous programmes funded by APIS-GENE and the French National Agency for Research have described new genes associated with growth potential, beef tenderness or flavor ([1,2], for a review, see, [10]). One of these results concerning expression of the *DNAJA1* gene was patented [11]. The GENOTEND programme aimed to validate the relationship between expression of these genes and beef quality attributes, including tenderness and flavor. To achieve this objective, we designed an Agilent chip with specific probes for the bovine muscular genes known to be involved in muscle growth (including energy and protein metabolism), carcass composition, fat metabolism and beef quality (including marbling). Our objective here was to confirm the use of this new genomic tool to predict meat quality by looking at the previously identified relationships ([12]; for a review, see, [10]) between beef tenderness and expression of genes belonging to two families (heat shock proteins and energy metabolism).

## Methods

### Design of the GENOTEND chip

More than 3000 genes involved in muscle biology or meat quality were selected from genetic programmes (for instance, [13], about 25% of the selected genes), proteomic studies (for instance, [14], for a review, see [15] ; about 14% of the selected genes), transcriptomic studies (for instance, [1,2,12], more than 30% of the selected genes), or from scientific publications (many genes have been previously listed by [7,9,10], about 20% of the selected genes). Other genes related to muscle biology were selected on the basis of the authors' expert knowledge. The list of publications from which genes were selected is indicated in Table 1.

**Table 1:** Main references for the selected genes in the GENOTEND chip

**Gene families**	**Main references**	**Proportion (%)**
Muscle hypertrophy and growth, differences between muscles, beef quality especially tenderness (our own transcriptomic studies)	[1] Meat Sci., 82, 205-212; [2]. Meat Sci., 70, 267-277; [12]. J. Agric. Food Chem., 55, 5229-5237; [16]. EAAP 112, 371-377; [17]. J. Physiol. Pharmacol., 60, Suppl 2, 83-90; [18]. J. Biochem. 133, 745-756 and unpublished results	33
Muscle fœtal ontogenesis, muscle hypertrophy and growth, beef quality (especially tendernesss) and *post-mortem* ageing (our own proteomic studies)	[14]. Proteomics 5, 490-500; [19]. Proteomics 6, 2571-2575; [20]. Proteomics 8, 4236-4248; [21]. Animal 3, 980-1000; [22]. Animal, 6, 867-874; [23]. Meat Sci., 78, 297-304.	14
Muscle hypertrophy and growth, intramuscular fat deposition, beef quality especially tenderness (French, European and international genetic studies)	[9]. Animal, 1, 159-173; [13]. Anim Genet, 40, 486-491; [24]. J. Anim. Sci., 89, 1-11; [25]. Animal, 4, 303–319; [26]. J. Anim. Sci., 85, 2660-2669 and unpublished results	25
Muscle hypertrophy and growth, differences between muscles, intramuscular fat deposition, beef quality (Australian studies)	[27]. J Anim Sci 84, 3239-3250; [28]. BMC Dev. Biol., 7,95; [29] Mamm. Genome 16, 201–210; [30] Aust. J. Exp. Agr. 45, 809–820; [31] J. Anim. Sci. 87, 119–130.	7
Muscle hypertrophy and growth, differences between muscles, intramuscular fat deposition, beef quality and ageing (American and other European studies)	[8]. Meat Sci., 74, 3-16; [32]. 83, 2075-2086; [33]. 6, 2720-2731; [34]. 5, 1763-1769; [35]. Meat Sci., 81, 731-737; [36]. Meat Sci., 85, 515-518; [37]. Anim Genetics 34, 438-444.	12
Muscle biology	Expertise of the authors	9

Whenever possible, several probes were used for each gene (e.g., 17 probes for *DNAJA1*). On average, four to five different probes were designed for each gene of interest.

### Animals and samples

This study took place with two groups of 25 and 30 Charolais young bulls and two groups of 21 and 19 Charolais steers (castrated at 3 months of age) slaughtered in two different years (years 1 and 2) from the French MUGENE programme (Genanimal-APISGENE 2005-2009). All animals proceed from the same experimental farm (Bourges) and were progeny of the same seed stock sample. The young bulls were reared in the same experimental farm (Bourges) and slaughtered at 17 months on average. The steers were reared in the experimental farm “Le Pin au Haras” and slaughtered at 30 months on average. All animals were slaughtered in the same conditions and without any electrical stimulation at the INRA experimental slaughterhouse in compliance with current INRA ethical guidelines for animal welfare [22]. Muscle samples from the *Longissimus thoracis* were excised within 15 minutes after slaughter. Muscle samples were immediately frozen in liquid nitrogen and stored at -80°C until RNA extraction.

In addition, steaks for each muscle were aged at 4°C for 14 days, and then frozen at -20°C. They were thawed rapidly under flowing water. On the following day they were grilled to a core temperature of 55°C and immediately served to trained panellists who scored initial tenderness and overall tenderness on 10-point scales: from 1 (tough) to 10 (tender). A total of 10-12 trained panelists were used in each session. Panelist underwent 8-10 test sessions for training, and during these they evaluated meat from different muscles, type of animals, and cooking processes [12].

### Hybridization studies

After extraction, total RNA was quantified with a Nanodrop ND.1000 spectrophotometer (Thermo Scientific, World Headquarters Location, Waltham, USA). RNA integrity was evaluated with the 2100 bioanalyzer (Agilent Technologies, Massy, France) and the RNA 600 Lab Chip kit. The total RNA was amplified and labeled with Cyanine 3 using Agilent’s Low RNA Input Linear Amplification Kit, PLUS, One-Color (Agilent Technologies) following the detailed protocol described by Agilent. Briefly, 500 ng of total RNA was reverse transcribed to double-strand cDNA using a poly dT-T7 promoter primer. cDNA products were then used as templates for in vitro transcription to generate fluorescent cRNA. Labeled cRNAs were finally purified using QIAGEN’s RNeasy mini spin columns and eluted in 30 μl of nuclease-free water. After amplification and labeling, cRNA quantity and cyanine incorporation were determined using a Nanodrop ND.1000 spectrophotometer (Thermo Scientific). For each hybridization, 600 ng of Cyanine 3 labeled cRNA were fragmented and hybridised at 65°C for 17 hours to an Agilent 8 x 15 K custom Oligo Microarray. After washing, microarrays were scanned using an Agilent DNA G2505B scanner. Feature extraction 9.1 software (Agilent Technologies) was used to assess fluorescent hybridization signals.

### Statistical analyses

The total number of probes was 10257, of which 1614 were control probes used for normalization. After data extraction with Feature Extraction (Agilent Technologies), hybridization values were averaged for replicated probes. Generally, probes for the same gene gave similar results. For each array, normalization was applied with the median of the 1614 control probes whose average expression level did not significantly differ between animal groups.

Normalization was then calculated per probe from the median of the probe obtained from all arrays. This ensured to use the same scale for all probes. Then a log2 transformation was done.

A Principal Component Analysis was performed with 70 genes belonging to the heat shock protein family in the group of young bulls slaughtered year 1 (the reference group) in order to identify the most correlated genes to initial and overall tenderness. Correlation coefficients of Pearson and of Spearman (relevant in this case due to the relative small number of animals per group) between gene expression levels and scores of initial or overall tenderness were calculated using the Statistical software (Stat soft, World Headquarters Location, Tulsa, USA). Multiple regression analyses were performed using the Statistical software (Stat soft) with the heat shock protein genes the most correlated to tenderness scores from young bulls of the reference group to test if these genes may predict variability of tenderness in the other groups of animals. Multiple regression analyses were also performed by combining gene expression levels and muscle biochemical characteristics which were previously shown to be associated with shear force (a measure of beef tenderness), namely lipid content, insoluble and total collagen contents and muscle fiber mean area [38]. The pooled relationship between muscle characteristics and gene expression with beef tenderness was calculated across animal groups after removing the gender and the year effects, i.e. by working on the deviation of each observation to the animal group muscle mean divided by the standard deviation for the group.

A similar approach was conducted with 150 genes belonging to fat and energy metabolism.

## Results

### Muscle characteristics

Sensory scores for the other animal groups are indicated Table 2. For the Charolais young bulls slaughtered year 1 (the reference group), initial tenderness scores were on average 5.45 (± 1.004) while overall tenderness scores were on average 5.00 (± 1.115) with a coefficient of variability of 18-20% (Table 2). Using the same experimental design, Guillemin et al. [38] observed that total lipid content, insoluble and total collagen contents as well as muscle fiber mean area were the only muscle biochemical traits which differ between classes of shear force (a measure of tenderness) for the *Longissimus thoracis* muscle, which confirmed previous results from Renand et al. [6]. These biochemical data were re-analysed by variance analysis. As known from the literature [39], we observed lower contents of intramuscular fat content in young bulls than in steers (21.2 vs. 34.3 mg/g wet tissue, P < 0.0001) and higher total collagen contents in young bulls than in steers (3.80 vs. 3.12 μg of OH-proline / mg dry matter, P < 0.0001). Collagen solubility did not differ between genders as previously observed [39] but significantly differed between years (19.41 *vs.* 25.60 in years 1 and 2, respectively, P < 0.04) as did mean area of muscle fiber (2680 *vs.* 3247 μm^2^ in years 1 and 2, respectively, P < 0.0001).

**Table 2:** Meat sensory scores for the different groups of animals

		**Initial tenderness**		**Overall tenderness**	
**Animal group**	**Number of animals**	**Mean ± SD**	**Min-max**	**Mean ± SD**	**Min-max**
Young bulls slaughtered year 1	25	5.45 ± 1.004	3.35–7.38	5.00 ± 1.115	3.11-7.10
Steers slaughtered year 1	21	5.82 ± 0.912	4.10-7.10	5.39 ± 0.949	3.55-6.89
Young bulls slaughtered year 2	30	4.64 ± 0.544	3.27-5.71	4.54 ± 0.621	3.04-5.49
Steers slaughtered year 2	19	4.54 ± 0.643	3.55-5.63	4.47 ± 0.793	3.23-6.05

Initial tenderness and overall tenderness were scored within a gender x year on 10-point scales: from 1 (tough) to 10 (tender).

### Relationships between beef tenderness scores and gene expression levels in the reference group

Following the Principal Component Analysis with data from young bulls slaughtered year 1, initial and overall tenderness scores were shown to be associated with the expression of some specific genes of the heat shock protein (HSP) family. Thanks to correlation analyses, expression levels of 6 genes were found to be negatively associated with beef tenderness (Table 3) with the highest correlation coefficients (r > 0.40). In particular, *DNAJA1* expression was negatively correlated with initial or overall beef tenderness (r = -0.48 to -0.59) in young bulls of the reference group as previously observed [12]. These 6 genes explain up to 49-50% of the total variability in tenderness in the reference group.

**Table 3:** Genes belonging to the heat shock protein family whose expression levels were the most correlated with initial or overall tenderness scores in the reference group (young bulls slaughtered year 1)

				**Correlation with initial tenderness**		**Correlation with overall tenderness**	
	**Animal type**	**Year**	**Number of animals**	**Person coefficient**	**Spearman coefficient**	**Person coefficient**	**Spearman coefficient**
Hsp27*	young bulls	year 1	25	-0.53	-0.50	-0.51	-0.50
***DNAJA1***	young bulls	year 1	25	-0.50	-0.59	-0.48	-0.59
***DNAJC3***	young bulls	year 1	25	-0.48	-0.49	-0.40	-0.49
***HspH1***	young bulls	year 1	25	-0.47	-0.47	-0.43	-0.47
***HspA8***	young bulls	year 1	25	-0.46	-0.50	-0.44	-0.50
***HspB1***	young bulls	year 1	25	-0.46	-0.43	-0.42	-0.42
*DNAJB5*	young bulls	year 1	25	-0.42	-0.45	-0.39	-0.49
*HspA6*	young bulls	year 1	25	-0.41	-0.40	-0.42	-0.37
*CRYAB*	young bulls	year 1	25	-0.40	-0.38	-0.40	-0.41

Among the 70 genes belonging to the heat shock protein family with probes on the GENOTEND chip, some genes were identified with the highest correlation coefficients with tenderness scores (they were the most associated with tenderness through the principal component analysis). Individual correlation coefficients (Pearson coefficient and Spearman coefficient) are indicated for each gene. Standard errors on the estimates of Pearson coefficients are between 0.18 and 0.19. Multiple regression analyses were performed with the combination of the first 6 genes (bold type) for young bulls slaughtered year 1. All together, these 6 genes explained up to 49-50% of the total variability in tenderness in the reference group.

*Probes for Hsp27 were determined on the basis of previously published proteomic studies whereas probes for other genes were determined on the basis of previous transciptomic studies.

With the same approach, five genes belonging to the energy metabolism family were also shown to be the most associated with beef tenderness (Table 4). We indeed observed that the gene encoding the adipocyte-fatty acid binding protein (*FABP4*), expressed only in intramuscular adipocytes (and hence a marker of intramuscular adipocyte numbers, [26]), fatty acid synthase (*FASN*), diacylglycerol O-acyltransferase gene (*DGAT2*) and *PPAR* gamma (a transcription factor regulating fatty acid synthesis) were positively associated with tender beef in young bulls slaughtered year 1. In addition, a gene involved in catabolism of fatty acid (hydroxyacyl-Coenzyme A dehydrogenize beta subunit (*HADH-B*, that catalyzes the last three steps of mitochondrial beta-oxidation of long chain fatty acids) was associated with the most tender beef in young bulls slaughtered year 1. All together, these 5 genes explain up to 66-71% of the total variability in tenderness in the reference group.

**Table 4:** Genes belonging to the energy and fat metabolism family whose expression levels were the most correlated with initial or overall tenderness scores in the reference group (young bulls slaughtered year 1)

				**Correlation with initial tenderness**		**Correlation with overall tenderness**	
	**Animal type**	**Year**	**Number of animals**	**Person coefficient**	**Spearman coefficient**	**Person coefficient**	**Spearman coefficient**
***FABP4***	young bulls	year 1	25	0.42	0.31	0.41	0.39
***PPARG***	young bulls	year 1	25	0.59	0.48	0.52	0.46
***DGAT2***	young bulls	year 1	25	0.61	0.51	0.57	0.56
***FASN***	young bulls	year 1	25	0.61	0.56	0.59	0.52
***HADHB***	young bulls	year 1	25	0.44	0.49	0.47	0.46

Among the 150 genes belonging to the energy and fat metabolism family with probes on the GENOTEND chip, five genes were identified with the highest correlation coefficients with tenderness scores (they were the most associated with tenderness through the principal component analysis). Individual correlation coefficients (Pearson coefficient and Spearman coefficient) are indicated for each gene. Standard errors on the estimates of Pearson coefficients are between 0.17 and 0.19. Multiple regression analyses were performed with the combination of these 5 genes for young bulls slaughtered year 1. All together, these 5 genes explained up to 66-71% of the total variability in tenderness in the reference group.

### Cross-validation of the identified tenderness markers

Five groups of probes (corresponding to four genes) belonging to the HSP family (Hsp27 encoded by the *HspB1* gene, identified from proteomic studies with the accession number Gene Bank ID: GI:71037405, *HspB1* Gene Bank ID: NM_013560.2, *DNAJA1* Gene Bank ID: NM_001015637.1, *HspH1* Gene Bank ID: NM_001075302.1, and *HspA8* Gene Bank ID: NM_174345.3) were found to be significantly expressed in all animals across genders and years of slaughter (which means expression levels differ from zero). In contrast, only two genes related to fat metabolism (*FASN* and *HADHB*) were found to be significantly expressed in all animals and were not validated for all animal groups. Therefore, we attempted to validate only the five groups of probes belonging to the HSP family in all animal groups.

Multiple regression analysis showed that the five selected groups of probes belonging to the HSP family explain 39 and 44% of initial and overall tenderness respectively in the reference group (young bulls slaughtered year 1) in which they were identified (Table 5). Furthermore, they also explained from 37 to 49% of the variability in initial and overall tenderness in young bulls slaughtered year 2 and in steers slaughtered year 1 (P < 0.08), but not in steers slaughtered year 2 for which only 20-23% of the variability in tenderness can be explained (not significant, Table 5). Regression analysis was found to be very similar in three animal groups (young bulls slaughtered years 1 and 2, steers slaughtered year 1). This result validates the relevance of this set of genes identified in one group of Charolais young bulls to predict beef tenderness at least in two other groups which differ from the reference population by only one factor (animal gender or environmental conditions reflected by different years of slaughter). However, when animals differed from the reference population both by gender (castration *vs.* intact males) and environmental conditions (slaughter year 2 instead of year 1) such as in the case of steers slaughtered year 2, the selected genes cannot be used any more to predict beef tenderness at a significant level.

**Table 5:** Validation in the different animal groups of the five sets of probes belonging to the heat shock protein family to predict variability in tenderness

			**Initial tenderness**		**Overall tenderness**	
**Animal group**	**Number of animals**		**R**^**2**^	**P value**	**R**^**2**^	**P value**
Young bulls slaughtered year 1	25	HSP gene expression levels	0.39	0.07	0.44	***0.04***
		Biochemical traits	0.08	0.75	0.08	0.78
Steers slaughtered year 1	21	HSP gene expression levels	0.45	0.08	0.49	***0.05***
		Biochemical traits	0.42	0.06	0.41	0.06
Young bulls slaughtered year 2	30	HSP gene expression levels	0.37	***0.04***	0.40	***0.03***
		Biochemical traits	0.04	0.88	0.08	0.69
Steers slaughtered year 2	19	HSP gene expression levels	0.20	0.68	0.23	0.58
		Biochemical traits	0.19	0.52	0.21	0.49

Multiple regression analyses were performed with the combination of the 5 groups of probes (*DNAJA1HspH1, HspA8, plus two groups of probes for HspB1*-Hsp27) to predict beef initial or overall tenderness for young bulls or steers slaughtered year 1 or year 2. Multiple regression analyses were also performed with the combination of four muscle biochemical traits (Intramuscular fat content, mean fiber area, total collagen content and collagen solubility) previously shown [38] to be associated with beef quality. The proportion of the variability in tenderness which is explained by the combination of the 5 groups of probes is indicated (R^2^) as well as the associated P value.

With the same approach, we observed that the four muscle biochemical traits (intramuscular fat content, total and insoluble collagen contents and muscle fiber area), which were previously identified to differ between shear force classes in the *Longissimus thoracis* of the same animals [38], explained only 8% of the variability in tenderness in young bulls slaughtered year 1 (not significant). As well, they explained only 4-8% and 19-21% in young bulls and steers respectively slaughter year 2 (not significant). However, they could explain 42% of the variability in tenderness in steers slaughtered year 1 (P = 0.06, Table 5).

When the three animal groups in which the HSP genes were validated were analysed together after removing the gender and the year effects (namely a total of 76 animals: young bulls of years 1 and 2, steers of year 1, Table 6), this set of genes explained 20% of the variability in the initial and overall tenderness (P = 0.008), whereas the biochemical muscle traits known to be associated with tenderness (namely intramuscular fat content, mean fiber area, total collagen content and collagen solubility according to [38]) explained only 5-6% of the variability in the initial and overall tenderness (not significant, Table 6).

**Table 6:** Validation across genders and environmental conditions of the set of five groups of probes belonging to the heat shock protein family to predict variability in tenderness

		**Initial tenderness**		**Overall tenderness**	
**Predictors of tenderness**	**Number of animals**	**R**^**2**^	**P value**	**R**^**2**^	**P value**
Expression levels of *HspB1(*Hsp27), *DNAJA1, HspH1, HspA8*	76	0.20	***0.008***	0.20	***0.008***
Intramuscular fat content, mean fiber area, total collagen content and collagen solubility	76	0.06	0.39	0.05	0.49
Intramuscular fat content, mean fiber area, total collagen content and collagen solubility plus expression levels of *HspB1(*Hsp27), *DNAJA1, HspH1, HspA8*	76	0.27	***0.01***	0.25	***0.02***

Multiple regression analyses were performed with the combination of the 5 groups of probes (Hsp27, *DNAJA1, HspH1, HspA8, plus two groups of probes for HspB1-Hsp27*) to predict beef initial or overall tenderness for young bulls slaughtered year 1 (the reference group) or year 2 or steers slaughtered year 2. The four muscle biochemical traits (Intramuscular fat content, mean fiber area, total collagen content and collagen solubility) previously shown to be associated with beef quality [38] were analysed with a similar approach alone or in association with the 5 groups of probes (*DNAJA1, HspH1, HspA8, plus two groups of probes for HspB1-*Hsp27). The pooled relationship between muscle characteristics and gene expression with beef tenderness was calculated across animal groups after removing the gender and the year effects, i.e. by working on the deviation of each observation to the animal group muscle mean divided by the standard deviation for the group. The proportion of the variability in tenderness which is explained in the different situations is indicated (R^2^).

## Discussion

Of all the beef quality traits, tenderness is considered to be the most important. It appears that among the main determinants of tenderness are collagen characteristics and the muscle fiber type [6] and, in association with this, the extent of proteolysis during *post-mortem* ageing and also the amount of intramuscular fat which facilitates mastication of beef. Indeed, with the same animal groups, intramuscular fat content and collagen solubility were shown to be positively associated with tenderness, whereas muscle fiber cross-sectional area were shown to be negatively associated with tenderness [38]. However, the contribution of collagen characteristics to tenderness may depend on the cooking temperature (55°C in our work, which is lower than in other studies) since heat-induced changes of intramuscular connective tissue were observed [40]. Among the proteolytic systems which control *post-mortem* proteolysis [41], it has been recently proposed that the apoptotic pathway may be of great importance, since it is the first event occurring after the animal's death. Our results confirm these observations using a new DNA chip. The aim of this manuscript was hence to validate this tool by confirming two well-accepted or recently discovered biological determinants of beef tenderness: the relationships of beef tenderness scores (i) with expression levels of genes corresponding to heat shock proteins on one hand and (ii) with expression levels of genes related to fat and energy metabolism in muscle fibers on the other hand.

### Relationships between beef tenderness scores and expression levels of genes encoding heat shock proteins

After slaughter of the animals, there is generalized cell death in all organs and tissues due to the lack of oxygen and hence anoxia of all cells. This stage precedes *rigor-mortis* and subsequently meat ageing during which protein degradation occurs thanks to the activity of different proteolytic systems (for a review, see [42]). More and more evidence is now described in the literature indicating that the muscle death process at the beginning of conversion of muscle into meat may play an important role in regulating meat quality [41]. This cell death process, often referred to as apoptosis, is regulated by many proteolytic enzymes including caspases, the activity of these enzymes being itself regulated by many factors.

Cell death induces considerable cell stress and, in response to stress, cells rapidly produce heat shock proteins (HSP) that play a universal role in maintaining cellular homeostasis. HSP help in maintaining the integrity of the cell and have an anti-apoptotic activity [43]. For instance, proteomic studies showed that Hsp27 in fresh Blonde d’Aquitaine muscle and levels of Hsp27 fragments in aged meat explained up to 91% of variation in sensory scores [23]. It was hypothesized that higher levels of Hsp27 associated with limited aggregation of muscle proteins could facilitate the action of proteolytic enzymes during meat ageing. Therefore, the positive correlation between tenderness scores and levels of specific Hsp27 protein isoforms or fragments in Blonde d’Aquitaine [23] contrasts with the negative correlation we observed in this work between Hsp27 mRNA levels and tenderness scores. This may be explained by the different sources of variability between the two studies: ageing time [23] or animal variability in this study. However, our results fit well with the positive correlation of Hsp27 protein level and shear force value in Korean cattle [44]. Indeed, a recent study with French breeds confirms that correlation of Hsp27 level may be positive or negative depending on the breed [45]. Other authors have described the complex effects of meat pH upon the sub cellular distribution of muscle HSP during ageing of beef [46]. In other experiments, *HspB1* (encoding Hsp27) and its regulator genes were shown to be negatively correlated with intramuscular fat content while being associated with a high shear force [47] and hence low tenderness as in our work.

Our results with the GENOTEND chip also confirm the negative correlation between *DNAJA1* expression and the tenderness score [12], but for young bulls and steers of year 1 only, which confirms our hypothesis that some markers of beef quality are highly dependent on rearing practices and environmental conditions. *DNAJA1* encodes a member of the large 40 kDa heat shock protein family (Hsp40). This protein is a co-chaperone of the 70 kDa heat shock protein (Hsp70) and is believed to play a role in protein folding and mitochondrial protein import. The *DNAJA1*/Hsp70 complex also directly inhibits programmed cell death (or apoptosis), which supports the hypothesis of Ouali et al. [41] that apoptosis is important for the tenderization of beef during ageing. Depending on the animal group (young bulls or steers slaughtered year 1), *DNAJB9DNAJC3* or *DNJAC10*, which are also members of the Hsp40 family, were negatively correlated to tenderness scores (data not shown) just like *DNAJA1*, which supports the role of members of the Hsp40 family in tenderness. In humans, the DNAJ family has over 40 DNAJ members [48] and can be subdivided into three subfamilies: DNAJA proteins, DNAJB proteins and DNAJC proteins with different structural features. Our results also support the role of some HspA (Hsp70), and some HspB (small HSPs) including Hsp27, in addition to that of DNAJA1 (Hsp40). For instance, CRYAB, which encodes αB-crystallin and shares homology with Hsp27, was also negatively correlated with tenderness in steers. However, the precise genes of this family which are individually negatively correlated to tenderness scores depends on the animal group and hence on its characteristics (gender, breeding management, etc).

Nevertheless, a major finding of our study is that a subset of genes belonging to the heat shock protein family may be suitable to better explain than muscle biochemical characteristics the variability of tenderness scores for both two groups of Charolais animals of the same gender or reared in similar environmental conditions. However, when the animals differ from the reference population by more than one factor (gender, or environmental conditions, etc), the expression levels of these genes cannot explain anymore the variability of tenderness scores. Further work is thus needed to extent the list of gene markers to have a more generic prediction of tenderness in other conditions of rearing and/or slaughtering and, if possible, in other breeds.

### Relationships between beef tenderness scores and expression levels of genes related to muscle metabolism

As previously demonstrated recently by Zhao et al. [49], we observed that some genes known to be expressed only in adipocytes (*FABP4*), or involved in lipid deposition (*PPARG, DGAT2, FASN*) were characterized by expression levels positively associated with tenderness in young bulls slaughtered year 1. This strongly suggests that intramuscular fat level contributes to tenderness in agreement with previous research. Indeed, whereas it is well-known that intramuscular fat level affects mainly juiciness and flavor of beef, it also affects indirectly tenderness [50]. In addition, with the same samples of this study, Guillemin et al [38] observed that intramuscular fat content was 45% higher in the most tender meat samples (determined from the shearforce measurement) compared to the least tender meat samples. Intramuscular fat is mainly stored within intramuscular adipocytes embedded in a connective tissue matrix in close proximity to a blood capillary. So, in fat-rich beef, intramuscular fat deposited between fibers fascicules may disrupt the structure of muscle connective tissue, thus contributing to increase meat tenderness [51]. This is important in beef meat, in which the abundance and solubility of collagen (the main component of connective tissue) are a source of variation in tenderness especially in young bulls. Generally, intramuscular fat has a diluting effect on connective tissue (known to increase toughness) and consequently a tenderizing effect on meat. Meat from animals with a high intramuscular fat level often has a low shear force [47], and hence high tenderness. Therefore, it is not surprising to observe that *FABP4* expression level, known to be a marker of intramuscular adipocyte number [26], was positively correlated with tenderness. Similarly, the more fatty acids are stored in these adipocytes, the bigger the adipocytes are, which favors tenderness by diluting connective tissue. Storage of fatty acids is controlled by two mechanisms. The first is due to the action of *LPL* which hydrolyses circulating triglycerides and hence favors uptake of resulting fatty acids. The second is associated with i) the activity of *FASN* which is a key enzyme involved in the *de novo* fatty acid synthesis and more precisely the elongation of fatty acids and (ii) the activity of *DGAT* which catalyses the formation of triglycerides from diacylglycerol and Acyl-CoA.

It is also well-known that muscles composed of fast fibers which low intramuscular fat content are more susceptible to early *post-mortem* proteolytic degradation than muscles mainly composed of slow fibers. Fast fibers tend to have higher levels of stored glycogen (which favors pH decline during ageing) and higher amounts of Ca2 + -activated myosin ATPase (which favors the speed of ageing). Therefore, after a short period of ageing, we expect fast muscles to generate tender beef compared to slow muscles due to a faster rate of ageing (for a review, see [52]). However, this may be not true after 14 days of ageing when a significant part of differences in ageing speed have disappeared. This may explain why the proportion of slow fibers has been reported to improve tenderness in cattle, especially for *Longissimus* muscle although this relationship may be not true for other muscle types or not always valid with data across muscle types (for a review, see [53]). However, the observation that oxidative fibers are associated with tender beef has been confirmed in our study by a higher expression of the *HADHB* known to be mainly expressed in oxidative fibers and also in tender beef in our case.

In contrast to the situation with HSP genes, we found no subset of genes related to fat metabolism or muscle fiber metabolism which were significant across environmental conditions and genders to predict beef tenderness. In some ways, this observation confirms results recently obtained by de Jager et al. [54] who observed differences in expression of the lipid storage genes between tough and tender genotypes, but also observed large differences in the expression of genes involved in fat and energy metabolism across two different experimental sites in Australia independently of genotype. A likely explanation is that these gene expression changes are reflecting differences in metabolic status of the animals such as turnover rates of nutrients (fatty-acid precursors such as glucose, triglycerides and fatty acids) or mitochondrial activity, especially considering nutritional regimens, feed density, ambient temperature, slaughtering conditions, etc between the different experimental sites or the different years of slaughtering.

## Conclusions

The GENOTEND chip was designed and validated by confirming known or recently discovered relationships between tenderness scores and expression levels of genes encoding heat shock proteins or related to muscle fiber metabolism. Furthermore, this transcriptomic analysis, focused on some muscle/meat quality markers, provided the means for analysis of these genes in different animal types (young bulls or steers) reared in different environmental conditions (corresponding to two years of slaughtering). We observed that numerous markers of beef tenderness can be identified but they are often specific to an animal type (steer or young bull) or to environmental conditions. However, these results confirm the idea that heat shock proteins (especially of the Hsp40 family) on the one hand [12,41] or metabolic enzymes on the other hand ([26]) may be potential good markers of beef tenderness. More specifically, a subset of genes belonging to the HSP family is able to explain a significant proportion of the variability of tenderness across genders and environmental conditions in three animal groups over four unlike some muscle biochemical characteristics yet known to be negatively or positively associated to shear force of beef.

## Authors’ contributions

JFH, CBC, VV and ICM designed the study and were highly involved in the conception of the GENOTEND chip. JFH, CBC, HL, GR and ICM were the key persons who selected all the genes involved in muscle biology or meat quality from different research programmes. They also collected the samples. VV and BJ processed the samples and analysed the data. The authors thank the INRA unit UE1206 (EASM, Le Magneraud, F-17700 Saint Pierre d’Amilly) for sensory analyses. All authors read and approved the final manuscript.

## References

[B1] Bernard C Cassar-Malek I Renand G Hocquette JF Changes in muscle gene expression related to metabolism according to growth potential in young bulls Meat Sci 2009 82 205 212 10.1016/j.meatsci.2009.01.012 20416758

[B2] Sudre K Cassar-Malek I Listrat A Ueda Y Leroux C Jurie C Auffray C Renand G Martin P Hocquette JF Biochemical and Transcriptomic analyses of two bovine skeletal muscles in Charolais bulls divergently selected for muscle growth Meat Sci 2005 70 267 277 10.1016/j.meatsci.2005.01.012 22063483

[B3] Pareek CS Smoczynski R Pierzchala M Czarnik U Tretyn A From genotype to phenotype in bovine functional genomics Brief funct genomics 2011 10 165 171 10.1093/bfgp/elr019 21628315

[B4] Hughes LM Bao J Hu ZL Honavar V Reecy JM Animal trait ontology: The importance and usefulness of a unified trait vocabulary for animal species J Anim Sci 2008 86 1485 1491 10.2527/jas.2008-0930 18272850 PMC2569847

[B5] Eggen A Hocquette JF Genomic approaches to economic trait loci and tissue expression profiling: application to muscle biochemistry and beef quality Meat Sci 2004 66 1 9 10.1016/S0309-1740(03)00020-2 22063926

[B6] Renand G Picard B Touraille C Berge P Lepetit J Relationships between muscle characteristics and meat quality traits of young Charolais bulls Meat Sci 2001 59 49 60 10.1016/S0309-1740(01)00051-1 22062505

[B7] Cassar-Malek I Picard B Bernard C Hocquette JF Application of gene expression studies in livestock production systems: a European perspective Aust J Exp Agr 2008 48 701 710 10.1071/EA08018

[B8] Mullen AM Stapleton PC Corcoran D Hamill RM White A Understanding meat quality through the application of genomic and proteomic approaches Meat Sci 2006 74 3 16 10.1016/j.meatsci.2006.04.015 22062712

[B9] Hocquette JF Lehnert S Barendse W Cassar-Malek I Picard B Recent advances in cattle functional genomics and their application to beef quality Animal 2007 1 159 173 10.1017/S1751731107658042 22444219

[B10] Hocquette JF Cassar-Malek I Bernard C Picard B Functional genomics and new markers for beef production Anim Sci Pap Rep 2009 27 273 280

[B11] Bernard C Cassar-Malek I Hocquette JF Genomic marker for meat tenderness Patent 2006 06 300943.5. 12 September 2006, WO/2005/052133

[B12] Bernard C Cassar-Malek I Le Cunff M Dubroeucq H Renand G Hocquette JF New indicators of beef sensory quality revealed by expression of specific genes J Agr Food Chem 2007 55 5229 5237 10.1021/jf063372l 17547415

[B13] Williams JL Dunner S Valentini A Mazza R Amarger V Checa ML Crisà A Razzaq N Delourme D Grandjean F Marchitelli C García D Pérez Gomez R Negrini R Ajmone Marsan P Levéziel H Discovery, characterization and validation of single nucleotide polymorphisms within 206 bovine genes that may be considered as candidate genes for beef production and quality Anim Genet 2009 40 486 491 10.1111/j.1365-2052.2009.01874.x 19397516

[B14] Bouley J Meunier B Chambon C De Smet S Hocquette JF Picard B Proteomic analysis of bovine skeletal muscle hypertrophy Proteomics 2005 5 490 500 10.1002/pmic.200400925 15627970

[B15] Picard B Berri C Lefaucheur L Molette C Sayd T Terlouw C Skeletal muscle proteomics in livestock production Brief funct genomics 2010 9 259 278 10.1093/bfgp/elq005 20308039

[B16] Cassar-Malek I Ueda Y Bernard C Jurie C Sudre K Listrat A Barnola I Gentès G Leroux C Renand G Martin P Hocquette JF Hocquette JF, Gigli S Molecular and biochemical muscle characteristics of Charolais bulls divergently selected for muscle growth Indicators of milk and beef quality 2005 EAAP Publication 112, Wageningen Academic Publishers, Wageningen, The Netherlands 371 377

[B17] Cassar-Malek I Jurie C Bernard C Barnola I Micol D Hocquette JF Pasture-feeding of charolais steers influences skeletal muscle metabolism and gene expression J Physiol Pharmacol 2009 60 Suppl 2 83 90 19996487

[B18] Sudre K Leroux C Piétu G Cassar-Malek I Petit E Listrat A Auffray C Picard B Martin P Hocquette JF Transcriptome analysis of two bovine muscles during ontogenesis J Biochem 2003 133 745 756 10.1093/jb/mvg096 12869531

[B19] Chaze T Bouley J Chambon C Barboiron C Picard B Mapping of alkaline proteins in bovine skeletal muscle Proteomics 2006 6 2571 2575 10.1002/pmic.200500452 16493707

[B20] Chaze T Meunier B Chambon C Jurie C Picard B In vivo proteome dynamics during early bovine myogenesis Proteomics 2008 8 4236 4248 10.1002/pmic.200701101 18924180

[B21] Chaze T Meunier B Chambon C Jurie C Picard B Proteome dynamics during contractile and metabolic differentiation of bovine foetal muscle Animal 2009 3 980 1000 10.1017/S1751731109004315 22444818

[B22] Guillemin N Jurie C Cassar-Malek I Hocquette JF Renand G Picard B Variations in the abundance of 24 proteins biomarkers of beef tenderness according to muscle and animal type Animal 2011 6 867 874 22440028 10.1017/S1751731110002612

[B23] Morzel M Terlouw C Chambon C Micol D Picard B Muscle proteome and meat eating qualities of Longissimus thoracis of “Blonde d’Aquitaine” young bulls: A central role of HSP27 isoforms Meat Sci 2008 78 297 304 10.1016/j.meatsci.2007.06.016 22062282

[B24] Allais S Journaux L Levéziel H Payet-Duprat N Raynaud P Hocquette JF Lepetit J Rousset S Denoyelle C Bernard-Capel C Renand G Effects of polymorphisms in the calpastatin and μ-calpain genes on meat tenderness in three French beef breeds J Anim Sci 2011 89 1 11 10.2527/jas.2010-3063 21178177

[B25] Hocquette JF Gondret F Baéza E Médale F Jurie C Pethick DW Intramuscular fat content in meat-producing animals: development, genetic and nutritional control, identification of putative markers Animal 2010 4 303 319 10.1017/S1751731109991091 22443885

[B26] Jurie C Cassar-Malek I Bonnet M Leroux C Bauchart D Boulesteix P Pethick DW Hocquette JF Adipocyte fatty acid-binding protein and mitochondrial enzyme activities in muscles as relevant indicators of marbling in cattle J Anim Sci 2007 85 2660 2669 10.2527/jas.2006-837 17565066

[B27] Lehnert SA Byrne KA Reverter A Nattrass G Greenwood PL Wang YH Hudson NJ Harper GS Gene expression profiling of bovine skeletal muscle in response to and during recovery from chronic and severe undernutrition J Anim Sci 2006 84 3239 3250 10.2527/jas.2006-192 17093216

[B28] Lehnert SA Reverter A Byrne KA Wang Y Nattrass GS Hudson NJ Greenwood PL Gene expression studies of developing bovine longissimus muscle from two different beef cattle breeds BMC Dev Biol 2007 7 95 10.1186/1471-213X-7-95 17697390 PMC2031903

[B29] Wang YH Byrne KA Reverter A Harper GS Taniguchi M McWilliam SM Mannen H Oyama K Lehnert SA Transcriptional profiling of skeletal muscle tissue from two breeds of cattle Mamm Genome 2005 16 201 210 10.1007/s00335-004-2419-8 15834637

[B30] Wang YH Reverter A Mannen H Taniguchi M Harper GS Oyama K Byrne KA Oka A Tsuji S Lehnert SA Transcriptional profiling of muscle tissue in growing Japanese Black Cattle to identify genes involved with the development of intramuscular fat Aust J Exp Agr 2005 45 809 820 10.1071/EA05058

[B31] Wang YH Bower NI Reverter A Tan SH De Jager N Wang R McWilliam SM Cafe LM Greenwood PL Lehnert SA Gene expression patterns during intramuscular fat development in cattle J Anim Sci 2009 87 119 130 18820161 10.2527/jas.2008-1082

[B32] Lin CS Hsu CW Differentially transcribed genes in skeletal muscle of Duroc and Taoyuan pigs J Anim Sci 2005 83 2075 2086 16100062 10.2527/2005.8392075x

[B33] Jia XH Ekman M Grove H Faergestad EM Aass L Hildrum KI Hollung K Proteome changes in bovine longissimus thoracis muscle during the early post-mortem storage period J Proteome Res 2007 6 2720 2731 10.1021/pr070173o 17567165

[B34] Jia XH Hildrum KI Westad F Kummen E Aass L Hollung K Changes in enzymes associated with energy metabolism during the early post mortem period in Longissimus thoracis bovine muscle analyzed by proteomics J Proteome Res 2006 5 1763 1769 10.1021/pr060119s 16823984

[B35] Pannier L Sweeney T Hamill RM Ipek F Stapleton PC Mullen AM Lack of an association between single nucleotide polymorphisms in the bovine leptin gene and intramuscular fat in Bos taurus cattle Meat Sci 2009 81 731 737 10.1016/j.meatsci.2008.11.014 20416562

[B36] Pannier L Mullen AM Hamill RM Stapleton PC Sweeney T Association analysis of single nucleotide polymorphisms in DGAT1, TG and FABP4 genes and intramuscular fat in crossbred Bos taurus cattle Meat Sci 2010 85 515 518 10.1016/j.meatsci.2010.02.025 20416823

[B37] Potts JK Echternkamp SE Smith TPL Reecy JM Characterization of gene expression in double-muscled and normal-muscled bovine embryos Anim Gen 2003 34 438 444 10.1046/j.0268-9146.2003.01055.x 14687074

[B38] Guillemin N Jurie C Renand G Hocquette JF Micol D Lepetit J Levéziel H Picard B Different phenotypic and proteomic markers explain variability of beef tenderness across muscles Int J Biol 2012 4 26 38

[B39] Dikeman ME Reddy GB Arthaud VH Tuma HJ Koch RM Mandigo RW Axe JB Longissimus muscle quality, palatability and connective tissue histological characteristics of bulls and steers fed different energy levels and slaughtered at four ages J Anim Sci 1986 63 92 101 3733582 10.2527/jas1986.63192x

[B40] Chang HJ Xu XL Li CB Huang M Liu DY Zhou GH A comparison of heat-induced changes of intramuscular connective tissue and collagen of beef semitendinosus muscle during water bath and microwave heating J Food Process Eng 2010 34 2233 2250

[B41] Ouali A Herrera-Mendez CH Coulis G Becila S Boudjellal A Aubry L Sentandreu M Revisiting the conversion of muscle into meat and the underlying mechanisms Meat Sci 2006 74 44 58 10.1016/j.meatsci.2006.05.010 22062715

[B42] Kemp CM Sensky PL Bardsley RG Buttery PJ Parr T Tenderness – An enzymatic view Meat Sci 2010 84 248 256 10.1016/j.meatsci.2009.06.008 20374783

[B43] Park KM Pramod AB Kim JH Choe HS Hwang IH Molecular and biological factors affecting skeletal muscle cells after slaughtering and their impact on meat quality: a mini-review J Muscle Foods 2010 21 280 307 10.1111/j.1745-4573.2009.00182.x

[B44] Kim NK Cho S Lee SH Park HR Lee CS Cho YM Choy YH Yoon D Im SK Park EW Proteins in longissimus muscle of Korean native cattle and their relationship to meat quality Meat Sci 2008 80 1068 1073 10.1016/j.meatsci.2008.04.027 22063838

[B45] Picard B Chaze T Meunier B Renand G Journaux L Capel C Hocquette JF Beef tenderness markers in the three main French breeds: a proteomic analyses 2011 Proceedings of the BIT’s 2nd World DNA and Genome Day, Dalian, Chine 185

[B46] Pulford DJ Fraga Vazquez S Frost DF Fraser-Smith E Dobbie P Rosenvold K The intracellular distribution of small heat shock proteins in post-mortem beef is determined by ultimate pH Meat Sci 2008 79 623 630 10.1016/j.meatsci.2007.10.027 22063023

[B47] Kim NK Lim D Lee SH Cho YM Park EW Lee CS Shin BS Kim TH Yoon D Heat shock protein B1 and its regulator genes are negatively correlated with intramuscular fat content in the Longissimus Thoracis muscle of Hanwoo (Korean cattle) steers J Agr Food Chem 2011 59 5657 5664 21524092 10.1021/jf200217j

[B48] Kampinga HH Hageman J Vos MJ Kubota H Tanguay RM Bruford EA Cheetham ME Chen B Hightower LE Guidelines for the nomenclature of the human heat shock proteins Cell Stress Chaperones 2009 14 105 111 10.1007/s12192-008-0068-7 18663603 PMC2673902

[B49] Zhao C Tian F Yu Y Luo J Hu Q Bequette BJ Baldwin Vi RL Liu G Zan L Scott Updike M Song J Muscle transcriptomic analyses in Angus cattle with divergent tenderness Mol Biol Rep 2012 39 4185 93 10.1007/s11033-011-1203-6 21901422

[B50] Jeremiah LE Dugan MER Aalhus JL Gibson LL Assessment of the relationship between chemical components and palatability of major beef muscles and muscle groups Meat Sci 2003 65 1013 1019 10.1016/S0309-1740(02)00309-1 22063683

[B51] Nishimura T Hattori A Takahashi K Structural changes in intramuscular connective tissue during the fattening of Japanese black cattle: effect of marbling on beef tenderization J Anim Sci 1999 77 93 104 10064032 10.2527/1999.77193x

[B52] Lee SH Joo ST Ryu Y C.Skeletal muscle fiber type and myofibrillar proteins in relation to meat quality Meat Sci 2006 86 166 170 20605337 10.1016/j.meatsci.2010.04.040

[B53] Maltin C Balcerzak D Tilley R Delday M Determinants of meat quality: Tenderness Proc Nutr Soc 2003 62 337 347 10.1079/PNS2003248 14506881

[B54] De Jager N Hudson NJ Reverter A Cafe LM Greenwood PL Wang YH Pethick DW Barnard R Dalrymple BP Effects of tenderness genotypes and of experimental site on the expression of fat and ribosomal module genes 2010 Proceedings of the 9th World congress on Genetics Applied to Livestock Production, Leipzig, Germany

